# Global DNA Methylation in Cord Blood as a Biomarker for Prenatal Lead and Antimony Exposures

**DOI:** 10.3390/toxics10040157

**Published:** 2022-03-26

**Authors:** Yoshinori Okamoto, Miyuki Iwai-Shimada, Kunihiko Nakai, Nozomi Tatsuta, Yoko Mori, Akira Aoki, Nakao Kojima, Tatsuyuki Takada, Hiroshi Satoh, Hideto Jinno

**Affiliations:** 1 Faculty of Pharmacy, Meijo University, 150 Yagotoyama, Tempaku-ku, Nagoya 468-8503, Japan; okmt@meijo-u.ac.jp (Y.O.); 194331507@ccmailg.meijo-u.ac.jp (Y.M.); aakira@meijo-u.ac.jp (A.A.); kojiman@meijo-u.ac.jp (N.K.); 2 Health and Environmental Risk Research Division, National Institute for Environmental Studies, 16-2 Onogawa, Tsukuba 305-8506, Japan; iwai.miyuki@nies.go.jp; 3 Development and Environmental Medicine, Tohoku University Graduate School of Medicine, 2-1 Seiryo-machi, Aoba-ku, Sendai 980-8575, Japan; nakai-k@tokaigakuen-u.ac.jp (K.N.); nozomi@med.tohoku.ac.jp (N.T.); 4 Department of Pharmaceutical Sciences, Ritsumeikan University, 1-1-1 Nojihigashi, Kusatsu 525-8577, Japan; ttakada@ph.ritsumei.ac.jp; 5 Environmental Health Science, Tohoku University Graduate School of Medicine, Sendai 980-8575, Japan; h.satoh@med.tohoku.ac.jp

**Keywords:** global DNA methylation, global DNA hydroxymethylation, cord blood DNA, lead, antimony, birth cohort

## Abstract

DNA methylation is an epigenetic mechanism for gene expression modulation and can be used as a predictor of future disease risks. A prospective birth cohort study was performed to clarify the effects of neurotoxicants on child development, namely, the Tohoku Study of Child Development, in Japan. This study aimed to evaluate the association of prenatal exposure to five toxic metals—arsenic, cadmium, mercury, lead (Pb), antimony (Sb), and polychlorinated biphenyls (PCBs, N = 166)—with global DNA methylation in umbilical cord blood DNA. DNA methylation markers, 5-methyl-2′-deoxycytidine (mC) and 5-hydroxymethyl-2′-deoxycytidine (hmC), were determined using liquid chromatography-tandem mass spectrometry. The mC content in cord blood DNA was positively correlated with Pb and Sb levels (*r* = 0.435 and 0.288, respectively) but not with cord blood PCBs. We also observed significant positive correlations among Pb levels, maternal age, and hmC content (*r* = 0.155 and 0.243, respectively). The multiple regression analysis among the potential predictors demonstrated consistent positive associations between Pb and Sb levels and mC and hmC content. Our results suggest that global DNA methylation is a promising biomarker for prenatal exposure to Pb and Sb.

## 1. Introduction

Prenatal exposure to environmental chemicals, such as heavy metals and polychlorinated biphenyls, is a significant concern because it causes developmental abnormalities in neurological functions [1,2]. In addition, accurate estimation of cumulative exposure is crucial for studying the relationship between prenatal exposure to these chemicals and health outcomes. However, the accumulation period and excretion pattern of these chemicals differ in their physicochemical properties, complicating the estimation. Therefore, a universal exposure marker would help evaluate lifetime exposure levels and predict disease susceptibility and adult outcomes.

Epigenetic DNA methylation plays an essential role in fetal development, during which epigenetic marks of maternal and paternal genomes are edited drastically [3,4]. In addition, DNA methylation is involved in various health events, such as aging, cancer, and the etiology of cardiovascular disease [3,5,6,7]. Mammalian DNA methylation is regulated by the orchestrated activity of DNA methyltransferases (DNMT1 and DNMT3A/3B), methylating 2′-deoxycytidine at the C5 position to produce 5-methyl-2′-deoxycytidine (mC) [8]. DNMT1 mainly catalyzes DNA methylation at hemimethylated sites, where DNA methylation patterns in the parental strand are maintained in the newly replicated daughter strand. DNMT3A/3B mediates DNA methylation at hemi- and unmethylated sites. Methylated DNA in dinucleotide CpG islands of the promoter region recruits methyl-CpG-binding proteins as transcriptional suppressors to reduce gene expression activity. By contrast, DNA demethylation proceeds through both passive and active mechanisms. Passive depletion of mC occurs during semi-conserved DNA replication. Ten-eleven translocation (TET) oxygenases (TET-1/2/3) mediate active mC removal, producing 5-hydroxymethyl-2′deoxycytidine (hmC) and further oxidized products [9,10]. These oxidized bases are then erased from the DNA strands by base excision repair enzymes. Many epidemiological studies have reported the association of environmental exposures [11,12,13] with semi-global or site-specific DNA methylation patterns, but studies on global methylation in cord blood DNA are limited. Assuming that exogenous factors disturb epigenetic regulation during prenatal and early life periods, the resulting epigenetic insult may interrupt normal development and possibly change the susceptibility and risk of future diseases in offspring. Therefore, DNA methylation may serve as a candidate biomarker for future health outcomes [14].

The co-authors performed a birth cohort study called the Tohoku Study of Child Development (TSCD) in Japan. In the TSCD, hundreds of maternal-fetal matched samples, including those of umbilical cord blood, have been collected, and chemical exposure levels have been determined [15,16,17,18,19,20,21]. Therefore, the aim of this study was to assess the association of environmental exposure to five toxic metals, arsenic, cadmium, mercury, lead (Pb), antimony (Sb), and polychlorinated biphenyls (PCBs), with global DNA methylation in umbilical cord blood DNA.

## 2. Materials and Methods

### 2.1. Study Design, Subjects, and Sampling

**Figure 1:**
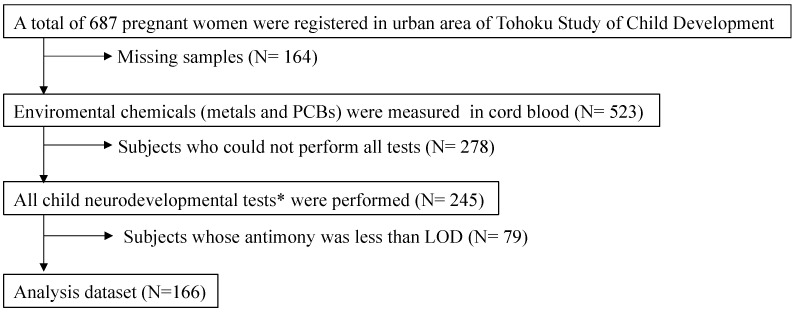
Flowchart of the study subjects. * NBAS, BSID-II, K-ABC, and WISC-III. LOD, limit of detection.

The study protocol for the TSCD was described [17]. The medical ethics committees of the Tohoku University Graduate School of Medicine (Approval number 2017-1-1032), National Institute for Environmental Studies (Approval number 2018-007), and Meijo University (Approval number 2012-22) approved the study protocol. Informed consent was obtained from all the participants. The TSCD was conducted in urban and coastal areas [19,22] and urban samples were used in this study (See [23] for detailed information of urban and coastal samples). We enrolled 687 women who provided written informed consent, and 599 mother–infant pairs were registered in March 2004 (participation rate: 87.2%). We selected subjects (*n* = 245) who completed data from all the developmental tests, such as NBAS (Neonatal Behavioral Assessment Scale), BSID-II (Bayley Scales of Infant and Toddler Development, the second edition), K-ABC (Kaufman Assessment Battery for Children), and WISC-III (Wechsler Intelligence Scale for Children, the third edition) for analysis. This study used 166 pairs with complete information on PCBs, mercury, lead, and other toxic element concentrations; global DNA methylation in cord blood; birth weight; possible confounders such as gestational age, parity, smoking and drinking habits during pregnancy; and maternal educational information (Figure 1).

### 2.2. Analytical Methods

#### 2.2.1. Determination of Toxic Metals and Essential Trace Elements

Total mercury (Hg, ng/g) in whole blood was measured using cold vapor atomic absorption spectrometry (CVAAS; HG-201, Sanso Seisakusho Co. Ltd., Tokyo, Japan). The analytical method for CVAAS has been described elsewhere [15,24]. We also determined the toxic metal levels of arsenic (As, ng/mL), cadmium (Cd, ng/mL), lead (Pb, μg/dL), and antimony (Sb, ng/mL), and the essential trace element levels of copper (Cu, ng/mL), zinc (Zn, ng/mL), and selenium (Se, ng/mL). These elements were measured using inductively coupled plasma mass spectrometry (7500c, Agilent Technologies, Inc., Santa Clara, CA, USA) [16].

Precision was ensured by using certified reference material (Seronorm Trace Elements Whole Blood L-2 and 3 prepared by SEROAS, Norway) for quality control. The data quality for the concentrations of these metals was validated using external quality assurance programs [25]. These analyses were performed by IDEA Consultants Inc. (Tokyo, Japan).

#### 2.2.2. Determination of Polychlorinated Biphenyls

All 209 PCB congeners were analyzed using high-resolution gas chromatography/high-resolution mass spectrometry with the isotope dilution method. The laboratory analytical methods and quality control procedures used have been described [18]. PCB analyses were performed by IDEA Consultants, Inc. (Tokyo, Japan). The quality of the PCB analyses was validated using an external quality assurance program, the German external assurance scheme at IDEA Consultants Inc. Accuracy was ensured by using a reference serum sample for quality control; the International Union of Pure and Applied Chemistry No. #28, #52, #101, #138, #153, #180 of PCB congeners were determined to be 0.310, 0.162, 0.150, 0.220, 0.217, and 0.300 μg/L, compared with the reference value (tolerance range in parenthesis) of 0.284 (0.181–0.388), 0.162 (0.102–0.222), 0.172 (0.126–0.218), 0.242 (0.174–0.310), 0.217 (0.152–0.281), and 0.307 (0.222–0.392) μg/L, respectively. Moreover, for cross-checking, PCBs were analyzed by another laboratory. PCB data from the two laboratories showed no significant difference by a paired *t* test (*t* = −2.572, *p* = 0.062) and a high Pearson product-moment correlation coefficient (*r* = 0.869). The calculated limit of detection (LOD) was 0.03 pg/g-wet, which was identified by the signal-to-noise ratio. We used the lipid basis for the total concentration of all measured PCB congeners (ΣPCBs), expressed as ng/g-lipid [26].

#### 2.2.3. Genomic DNA Extraction and Digestion

Genomic DNA extraction and enzymatic digestion were performed as previously reported [4,27,28]. Briefly, DNA was extracted from cord blood by using a spin column (High Pure PCR Template Preparation Kit; Roche, Mannheim, Germany) according to the manufacturer’s instructions. For enzymatic digestion, 1 μg DNA was incubated at 37 °C for 3 h with nuclease P1 (4U, Wako Pure Chemical Industries, Ltd., Osaka, Japan) and alkaline phosphatase (3U, Wako) in 100 μL of buffer mixture (30 mM sodium acetate (pH 5.3) containing 10 mM 2-mercaptoethanol and 20 mM ZnSO_4_), followed by the addition of 20 μL Tris-HCl (500 mM, pH8.5) and incubation for another 3 h. After methanol precipitation, the supernatant containing nucleosides was evaporated to dryness and stored at −80 °C until use. Each digest was reconstituted in 100 μL internal standard (IS) solution that contained the following stable isotope labeled standards, namely, 5-(methyl-*d_3_*)-2′-deoxycytidine (mC-*d_3_*, 0.1 nM; Toronto Research Chemicals Inc., Ontario, Canada), 5-(hydroxymethyl-*d_2_*)-2′-deoxycytidine (hmC-*d_2_*, 0.1 nM; synthesized in our laboratory [4]), and ^15^*N*_5_-2′-deoxyguanosine (*^15^N*_5_-G, 1 nM; Cambridge Isotope Laboratories Inc., Andover, MA, USA), and the aliquot was further diluted 10–100 times with IS solution before being subjected to LC-MS/MS. Calibration curves were obtained using the following unlabeled standards: 5-methyl-2′-deoxycytidine (Tokyo Chemical Industry Co., Ltd., Tokyo, Japan), 5-hydroxymethyl-2′-deoxycytidine (Berry and Associates Inc., Dexter, MI, USA), and 2′-deoxyguanosine (Sigma-Aldrich Co., St. Louis, MO, USA).

#### 2.2.4. LC-MS/MS Analysis for mC/hmC Quantification

LC-MS/MS analysis was performed using a high-performance liquid chromatograph (Prominence series, Shimadzu, Kyoto, Japan) equipped with a triple-quadrupole mass spectrometer (API4000 system, AB Sciex, Foster City, CA, USA) [4,28]. Sample aliquots (60 μL) were separated at 40 °C by using a reverse-phase column (TSKgel ODS-100 V, 4.6 mm × 75 mm × 3 μm; Tosoh, Tokyo, Japan) in the isocratic mode with a mobile phase (methanol-10 mM ammonium formate (20:80)) at a flow rate of 0.3 mL/min. Stable isotope labeled nucleosides were used as internal standards for mC, hmC, and G. Elution of mC, hmC, and G was, respectively, at 4.1, 5.0, and 5.3 min in the isocratic mode (total running time, 12 min). Mass spectral analysis was conducted in the positive ion mode with nitrogen as the nebulizing gas. Ionization was performed under the following conditions: curtain gas, 10 psi; collision gas, 8 psi; ion source gas 1, 60 psi; ion source gas 2, 60 psi; ion source voltage, 4500 V; and ion source temperature, 400 °C. Positive ions were acquired in the multiple reaction monitoring (MRM) mode. The MRM transitions were monitored as follows: mC (*m*/*z* 242→126), mC-*d_3_* (*m*/*z* 245→129), hmC (*m*/*z* 258→142), hmC-*d_2_* (*m*/*z* 260→144), G (*m*/*z* 268→152), and ^15^*N*_5_ g (*m*/*z* 273→157). The proportion (%) of C that was mC or hmC in each sample is given by %mC or hmC = [(value for mC or hmC)/(value for G)] × 100. The mC and hmC values were expressed as the content (ng) in 100 ng of DNA.

### 2.3. Statistical Analysis

Numerical variables were presented as mean ± standard deviation and as median and interquartile range. Categorical variables were presented as frequencies (percentages). The metal and PCB concentrations were logarithmically transformed because of a skewed distribution. Pearson product-moment correlation coefficients were used to determine the relationships among global DNA methylation, exposure biomarkers, and basal characteristics. A generalized linear model was used to evaluate the association between prenatal chemical exposure and DNA methylation. We selected potential covariates associated with at least one of the exposed substance-outcome pairs (i.e., maternal age, gestational age, BMI before pregnancy, smoking/drinking habits during pregnancy, education, delivery mode, and parity). We also created a directed acyclic graph [29] for covariate selection (Figure S1). The multicollinearity of these variables was less than the variance inflation factor five (VIF5). Because samples whose analytical values were less than the LOD were omitted (79 of 245 samples), the remaining 166 samples were subjected to statistical analysis. Sensitivity analysis confirmed a similar result for *n* = 245. Statistical significance was set at *p* < 0.05. Data analysis was performed using a software package (JMP16.0, SAS Institute Inc., Cary, NC, USA).

## 3. Results

The basal characteristics, exposure levels, and DNA methylation status of 166 mother–infant pairs are shown in Table 1. BMI (20.9 ± 2.5) indicated that the participants were classified as having a normal weight. The smoking rate during pregnancy (7.2%) was similar to that reported in the literature [30,31]. By contrast, 31.3% of mothers stated that they were drinking during pregnancy, and this value is several times higher than that reported (less than 10%) [32,33]. However, no substantial impairments in the infants were found in gestational age, birth weight, sex ratio, and Apgar score.

**Figure 2:**
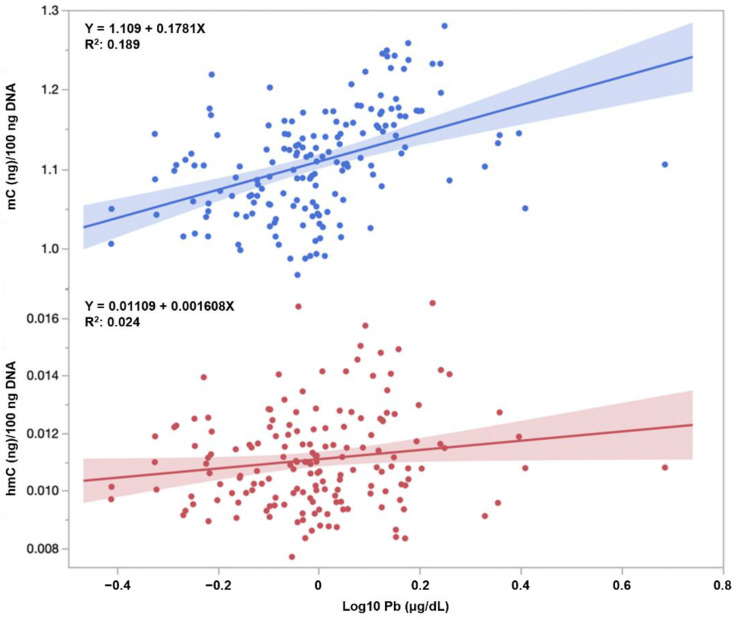
Relationship between cord blood Pb and mC content (upper) or hmC content (lower).

Table 2 shows the relationship between global DNA methylation, exposure biomarkers, and basal characteristics (*n* = 166, excluding samples with Sb levels less than the LOD). A positive correlation was found between Pb, Sb, and Se levels and mC content in cord blood (Pearson correlation coefficient, *r* = 0.435 for Pb, *r* = 0.288 for Sb, and *r* = 0.168 for Se; Figure 2). In addition, we observed a significant positive correlation between the hmC content and Pb level or maternal age (*r* = 0.155 for Pb and *r* = 0.243 for maternal age, Figure 2). By contrast, no significant differences in global DNA methylation were observed in smoking and drinking habits during pregnancy, although the population sizes were small.

The associations between the predictors and global DNA methylation are shown in Table 3. We found consistent positive associations between Pb, Sb, and Se levels and mC content in cord blood among potential predictors. In addition, cord blood Pb levels and maternal age were positively associated with the hmC content. These predictors explained 39.9% of the mC content in cord blood, whereas the hmC content was lower (18.1%).

## 4. Discussion

DNA methylation is an epigenetic mechanism for gene expression modulation and can be used as a predictor of future disease risks. Several large-scale studies have evaluated the association between prenatal chemical exposure and DNA methylation [34,35,36]. Most of those studies have measured the site-specific, not the global, DNA methylation status by using a bead-chip DNA microarray. The pyrosequencing technique is a powerful tool for estimating semi-global DNA methylation levels based on the methylation status in the LINE-1 retrotransposon [37]. Several groups have used this technique to study the relationship between chemical exposure and DNA methylation [38,39,40,41,42,43,44]. In this technique, unmethylated cytosine is distinguished from methylated cytosine based on bisulfite conversion, during which unmethylated cytosine is converted to uracil, and methylated cytosine reacts poorly with bisulfite. However, resistance to bisulfite conversion is also observed with hydroxymethylated cytosine, making it impossible to differentiate this base from methylated cytosine [45]. ELISA is another choice for determining global DNA methylation [46,47,48]. However, as epigenetic variations in cord blood DNA seem to be relatively low, the accuracy and precision of ELISA assays would be insufficient to assess epigenetic fluctuations during fetal development [49]. In this study, the LC-MS/MS method [2,18] was used for the first time for absolute quantification of mC and hmC levels in cord blood DNA.

The TSCD, whose aim was to evaluate the effect of prenatal exposure to environmental chemicals on neurological development, provided us with valuable cord blood samples. This study determined the association of prenatal exposure to five toxic metals (As, Cd, Hg, Pb, and Sb), three essential trace elements (Se, Cu, and Zn), and PCBs with the mC/hmC content in cord blood. Multiple regression analysis showed a statistically significant association between Pb or Sb exposure and DNA methylation (mC and hmC content) (Table 3). The association of LINE-1 methylation with maternal patellar Pb levels has also been shown [44]. Although blood Se level was weakly associated with mC content, Se level in serum/plasma, where Se-containing proteins exist, should be measured to understand the exact meaning of the association. Taken together, these findings and the previous report suggest that Pb exposure alters global DNA methylation; however, further studies are necessary.

The impact of Pb exposure on developmental cognitive functions has been extensively studied. In a meta-analysis of children’s IQ and blood Pb levels, Wu et al. reported that full-scale IQ scores dropped by 6.60 points in children with blood Pb levels ≥10 μg/dL [50]. In addition, several epidemiological studies have suggested no proven lower limit of Pb exposure, because a decrease in cognitive function has been observed in children with relatively low blood Pb concentrations (<10.0 μg/dL) [21,51,52]. Although the blood Pb levels in preschool children globally decreased to less than 6.0 μg/dL, the children suffer from Pb exposure, especially in countries with a medium/low UN human development index [53]. The Human Biomonitoring Commission of the Federal Environment Agency in Germany concluded that any setting of an “effect threshold” for blood Pb levels would be arbitrary and unjustified [54]. As a result, the Commission suspended the human biomonitoring values for Pb in the blood of children and adults. The United States Centers for Disease Control and Prevention also announced a reduction in the blood Pb reference value for children from 5 to 3.5 μg/dL in Oct. 2021 [55]. Nevertheless, there are no valuable biomarkers for estimating the impact of low lead exposure. This study found that global DNA methylation was strongly associated with cord blood Pb ranging from 0.39 to 4.84 μg/dL (Table 3). Our results suggest that global DNA methylation could be a promising biomarker for the adverse health effects of prenatal Pb exposure.

This study has limitations. First, although we found relationships between environmental chemicals, such as Pb and Sb, and global DNA methylation, we did not assess the methylation of individual genes and other chemicals. Second, the sample size was small, and Pb exposure was narrow and not high. Third, the uncontrolled confounding variables have remained in our analysis (Figure S1). Further studies with larger sample sizes and more comprehensive approaches than those used in this study are necessary to verify these results.

## 5. Conclusions

We determined the relationship between environmental chemical levels (As, Cd, Hg, Pb, Sb, and PCBs) and the mC/hmC content in cord blood DNA. The cord blood Pb and Sb concentrations were positively associated with mC and hmC content. Notably, the Pb exposure level was relatively low, ranging from 0.39 to 4.84 μg/dL. Although further studies are necessary, global DNA methylation could be a suitable biomarker for estimating Pb exposure and predicting future health outcomes.

## Figures and Tables

**Table 1:** Basal characteristics, exposure levels, and DNA methylation status in this study’s subjects.

*n* = 166	Mean ± SD Median (P25–P75)	*n* (%)
*Maternal characteristics*		
Maternal age (years)	31.2 ± 3.8	
Body mass index before pregnancy (kg/m^2^)	20.9 ± 2.5	
Smoking habit during pregnancy (smokers, %)		12 (7.2)
Drinking habit during pregnancy (drinkers, %)		52 (31.3)
Delivery type (spontaneous, %)		122 (73.5)
Parity (first, %)		88 (53.0)
Maternal educational level (graduate high school, %)		128 (77.1)
*Baby characteristics*		
Gestational age (weeks)	39.6 ± 1.3	
Birth weight (g)	3078.0 ± 329.1	
Sex (boys, %)		88 (53.0)
Apgar score	8 (8–9)	
*Exposure levels in cord blood*		
Total PCBs (ng/g-lipid)	49.6 (30.3–60.5)	
Hg (ng/g)	10.8 (7.0–13.7)	
As (ng/mL)	4.42 (2.69–5.52)	
Pb (μg/dL)	1.06 (0.80–1.27)	
Cd (ng/mL)	0.93 (0.05–1.06)	
Sb (ng/mL)	0.93 (0.41–1.28)	
Se (ng/mL)	185.6 (158.9–210.6)	
Cu (ng/mL)	512.8 (448.5–548.5)	
Zn (ng/mL)	2129.7 (1729.7–2236.6)	
*DNA methylation status*		
mC (ng/100 ng DNA)	1.11 (1.06–1.15)	
hmC (ng/100 ng DNA)	0.011 (0.010–0.012)	

**Table 2:** Pearson product-moment correlation coefficients (*r*) between relating indicators and mC/hmC contents (ng/100 ng DNA).

*n* = 166	mC (ng/100 ng DNA)	hmC (ng/100 ng DNA)
	*r*	*r*
*Maternal characteristics*		
Maternal age (years)	0.089	0.243 *
Body mass index before pregnancy (kg/m^2^)	−0.047	−0.072
*Baby characteristics*		
Gestational age (weeks)	0.033	−0.096
Birth weight (g)	0.052	−0.027
Birth length (cm)	0.128	0.067
*Exposure levels in cord blood*		
PCBs (ng/g-lipid)	−0.090	0.050
Hg (ng/g)	0.038	−0.074
As (ng/mL)	−0.058	−0.123
Pb (μg/dL)	0.435 **	0.155 *
Cd (ng/mL)	−0.010	−0.129
Sb (ng/mL)	0.288 **	0.125
Se (ng/mL)	0.168 *	0.008
Cu (ng/mL)	0.089	0.044
Zn (ng/mL)	0.036	−0.063

Values were Pearson product-moment correlation coefficients (*r*). * *p* < 0.05, ** *p* < 0.01.

**Table 3:** Relations of exposure levels and possible confounders to mC and hmC content (ng/100 ng DNA): Results of multiple regression analysis.

*n* = 166	mC (ng/100 ng DNA)		hmC (ng/100 ng DNA)	
	Standardized Regression Coefficient, *β* [95% CI]	*p* Value	Standardized Regression Coefficient, *β* [95% CI]	*p* Value
*Exposure markers*				
PCBs (ng/g-lipid)	−0.083 [−0.239, 0.073]	0.294	0.118 [−0.064, 0.300]	0.203
Hg (ng/g)	0.110 [−0.033, 0.253]	0.130	−0.053 [−0.219, 0.113]	0.530
As (ng/mL)	−0.085 [−0.224, 0.054]	0.228	−0.120 [−0.282, 0.042]	0.144
Pb (μg/dL)	0.524 [0.381, 0.666]	<0.0001	0.227 [0.061, 0.394]	0.008
Cd (ng/mL)	−0.056 [−0.195, 0.084]	0.430	−0.143 [−0.305, 0.020]	0.086
Sb (ng/mL)	0.388 [0.255, 0.521]	<0.0001	0.192 [0.037, 0.348]	0.016
Se (ng/mL)	0.153 [0.009, 0.297]	0.038	0.042 [−0.126, 0.211]	0.622
Cu (ng/mL)	−0.001 [−0.167, 0.164]	0.988	0.064 [−0.130, 0.257]	0.517
Zn (ng/mL)	−0.134 [−0.310, 0.042]	0.134	−0.194 [−0.400, 0.011]	0.064
*Possible confounders*				
Maternal age	0.093 [−0.055, 0.240]	0.216	0.176 [0.003, 0.348]	0.046
Gestational weeks	−0.031 [−0.172, 0.110]	0.665	−0.028 [−0.193, 0.137]	0.739
Parity	−0.020 [−0.178, 0.139]	0.808	0.057 [−0.128, 0.242]	0.545
Education levels	0.156 [−0.015, 0.326]	0.074	−0.128 [−0.327, 0.071]	0.207
BMI before pregnancy	−0.013 [−0.143, 0.117]	0.848	−0.057 [−0.209, 0.095]	0.457
Smoking habit during pregnancy	0.145 [−0.131, 0.421]	0.302	−0.052 [−0.374, 0.271]	0.752
Drinking habit during pregnancy	−0.046 [−0.184, 0.093]	0.516	−0.053 [−0.215, 0.109]	0.516
Contribution rate, *R*^2^ Adjusted *R*^2^	0.399 0.334		0.181 0.093	

*R* indicates the multiple correlation coefficient.

## Data Availability

Data are unsuitable for public deposition due to ethical restrictions and the legal framework in Japan. The Act on the Protection of Personal Information (Act No. 57 passed on 30 May 2003, amended on 9 September 2015) prohibits the public deposition of data containing personal information. Ethical Guidelines for Medical and Health Research Involving Human Subjects enforced by the Japan Ministry of Education, Culture, Sports, Science and Technology and the Ministry of Health, Labour and Welfare also restrict the open sharing of epidemiologic data. All inquiries about access to data should be sent to: Miyuki Iwai-Shimada.

## Supplementary Materials

The following supporting information can be downloaded at: https://www.mdpi.com/article/10.3390/toxics10040157/s1, Figure S1: Directed acyclic graph showing the hypothesized relationship among prenatal chemical exposure, DNA methylation, and covariates. Ht, Hematocrit value; v1, variable 1; v2, variable 2.
