# Supplementary material for: Global DNA Methylation in Cord Blood as a Biomarker for Prenatal Lead and Antimony Exposures
Source: Toxics. 2022 Mar 26;10(4):157. doi: 10.3390/toxics10040157 (PMC9027623; doi:10.3390/toxics10040157)
Supplement: Supplementary file 1 [file toxics-10-00157-s001.zip › toxics-1643735-supplementary.pdf]

toxics MDPI

Supplementary Materials: Global DNA Methylation in Cord Blood as a Biomarker for Prenatal Lead and Antimony Exposures

Yoshinori Okamoto, Miyuki Iwai-Shimada, Kunihiko Nakai, Nozomi Tatsuta, Yoko Mori, Akira Aoki, Nakao Kojima, Tatsuyuki Takada, Hiroshi Satoh and Hideto Jinno

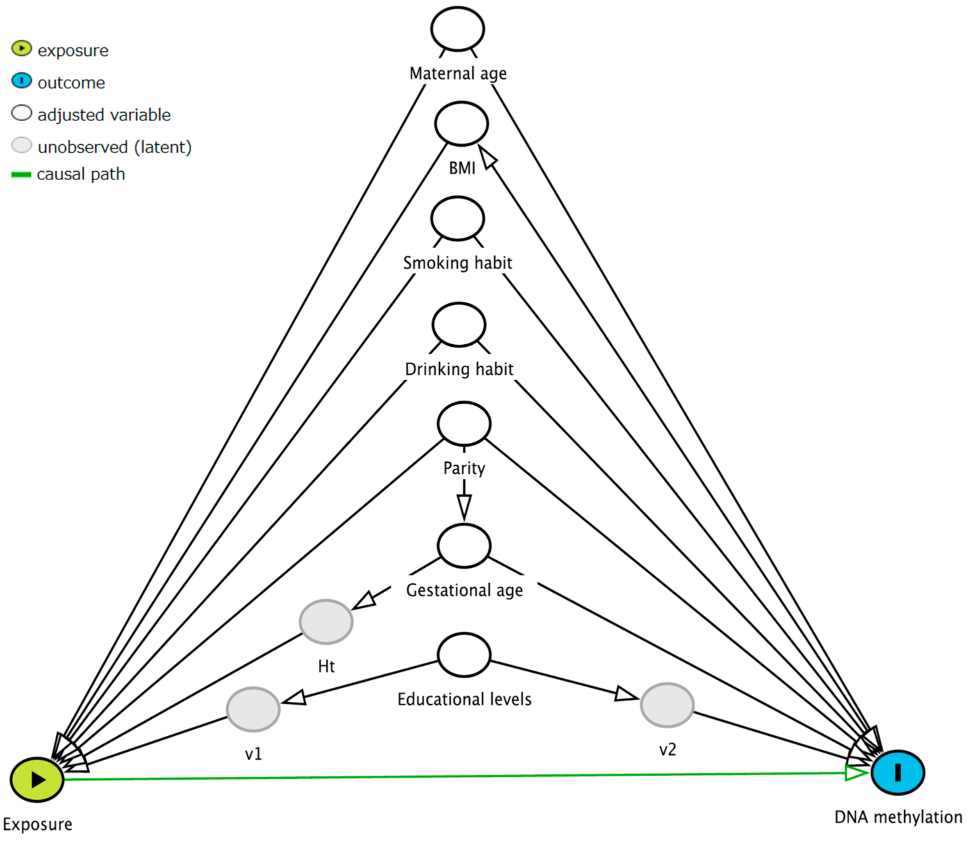

Figure S1. Directed acyclic graph showing the hypothesized relationship among prenatal chemical exposure, DNA methylation, and covariates. Ht, Hematocrit value; v1, variable 1; v2, variable 2.
